# Supplementary material for: Characterization of MET Alterations in 37 Gastroesophageal Cancer Cell Lines for MET-Targeted Therapy
Source: Int J Mol Sci. 2024 May 29;25(11):5975. doi: 10.3390/ijms25115975 (PMC11173193; doi:10.3390/ijms25115975)
Supplement: Supplementary file 1 [file ijms-25-05975-s001.zip › ijms-3037026-supplementary/Table S1.pdf]

Table S1. Primer sequence for droplet digital PCR

MMx no.	Gene	Probe sequence	Primer sequence	
**MMx1**	Mep15	CCATCCTAACTAGTGGGGACTCTGA	Forward	AAGTGCAGTATCCTCTGACAGACA
			Reverse	TGGACAGTATTTTGCAGTAATGG
	AP3B1	CACAGTTTGTCAGCCCTTGGA	Forward	CCACATGCTAACTCGATATGC
			Reverse	GTGGAAGACTGTTCAACACTTACC
**MMx2**	RnaseP	ACGGCCAGCGAAGTGAGTTC	Forward	TGCCGGAGCTTGGAACAG
			Reverse	AAAATGGGCGGAGGAGAGTAG
	EIF2C1	TCCAACCTCTGCCTCAAGATC	Forward	TGGTCAAGACCTCACCTCAG
			Reverse	TGTTAATGCCACCAAGTTTGAC
**MMx3**	RPP30	GGAATTGTCAAACTGACTCCTTTTCC	Forward	ATTTTAGGATGTTTTTCGCATCTG
			Reverse	TGGCAGAATTTGTTCCTATGC
	PPIA	TTGGATGGCAAGCATGTGGT	Forward	GTTGCCAGTCATAGTGATTGTTC
			Reverse	GGCCTCCACAATATTCATGC
