# Supplementary figures and images for: Characterization of MET Alterations in 37 Gastroesophageal Cancer Cell Lines for MET-Targeted Therapy
Source: Int J Mol Sci. 2024 May 29;25(11):5975. doi: 10.3390/ijms25115975 (PMC11173193; doi:10.3390/ijms25115975)

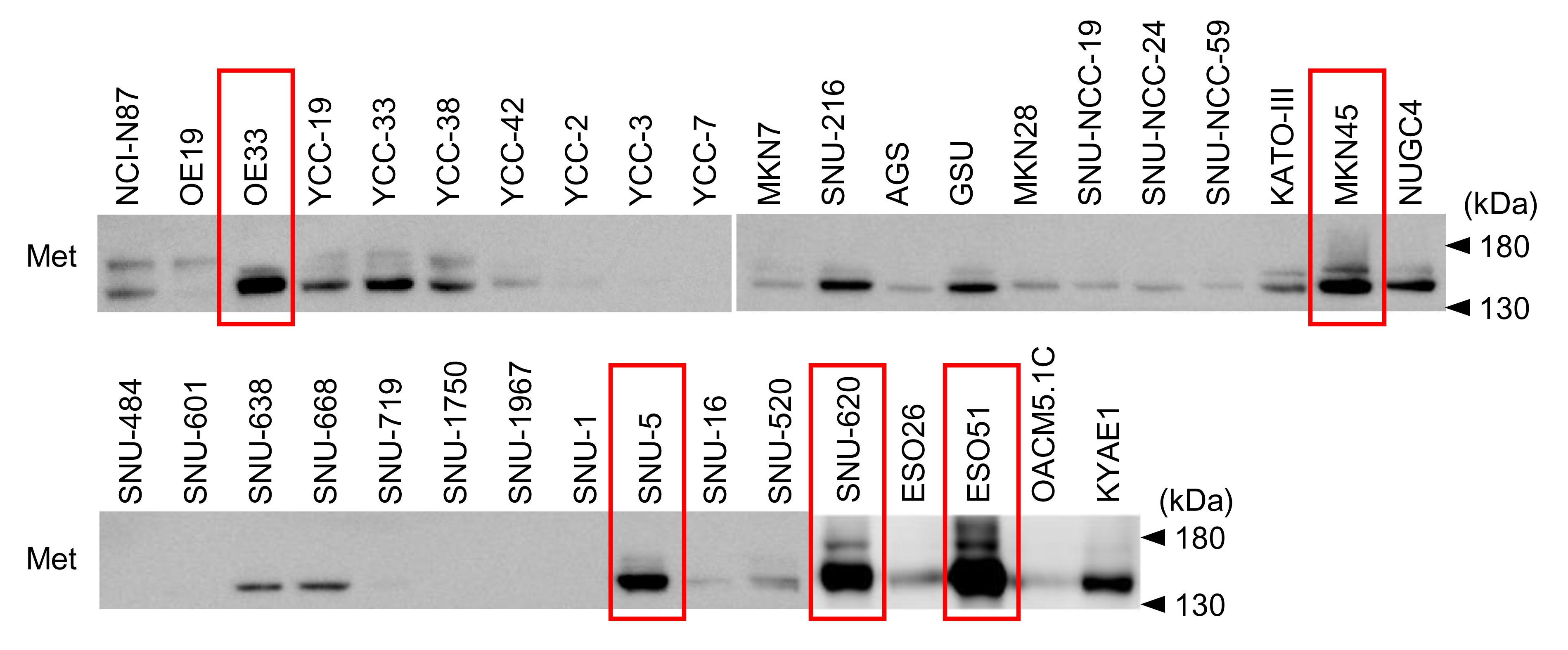

Supplement: Supplementary file 1 [file ijms-25-05975-s001.zip › ijms-3037026-supplementary/Figure S1.tif]

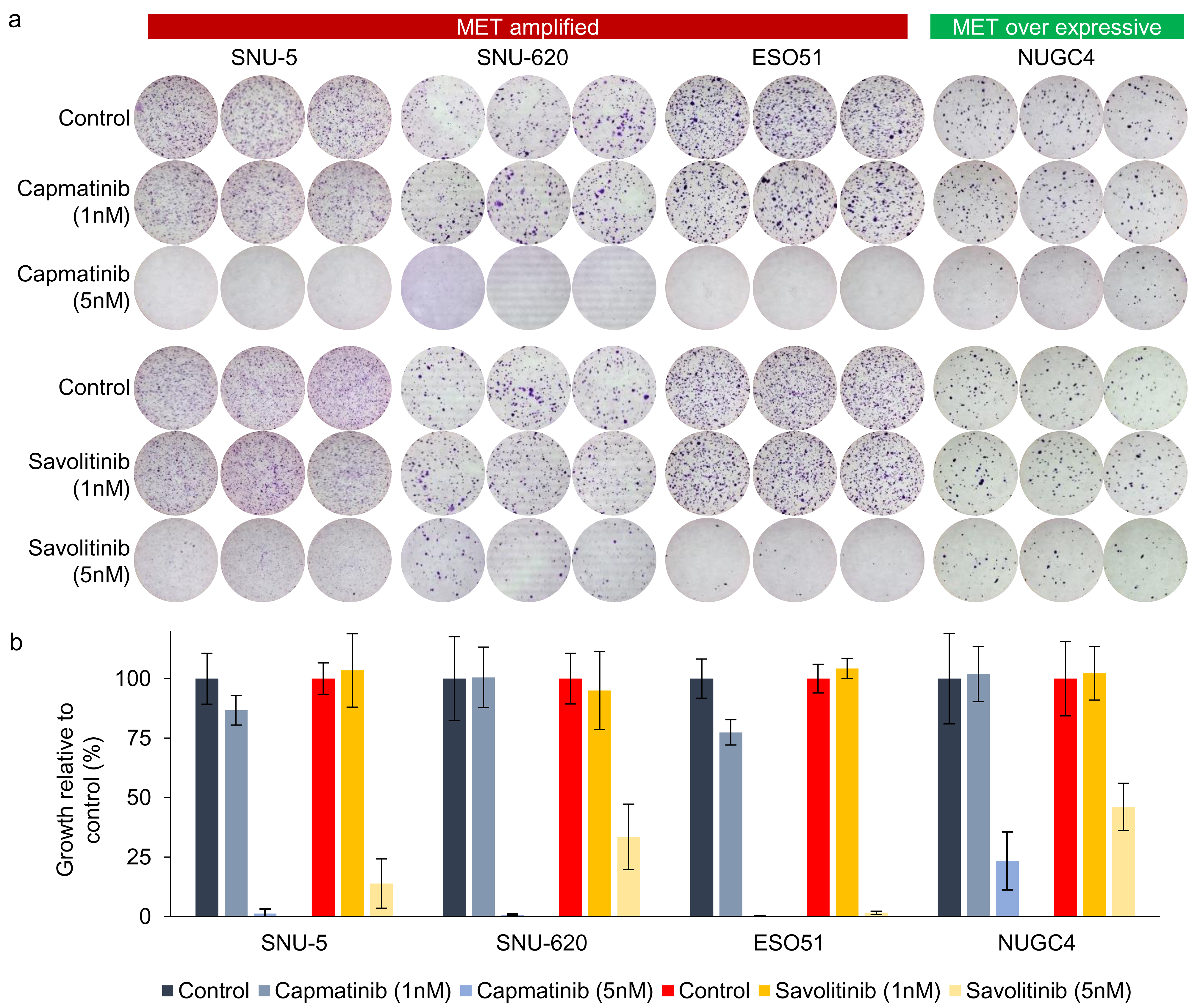

Supplement: Supplementary file 1 [file ijms-25-05975-s001.zip › ijms-3037026-supplementary/Figure S2.tif]

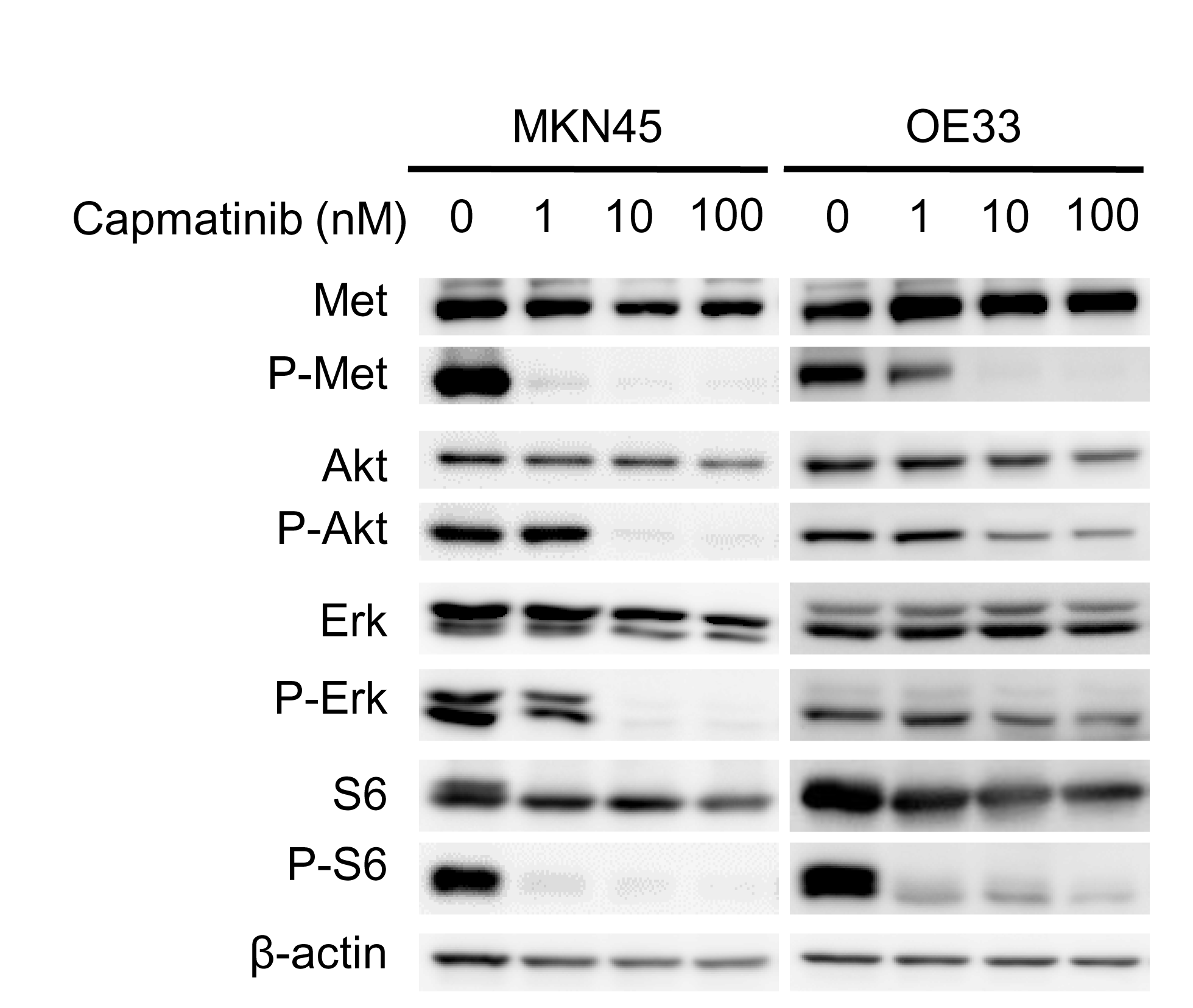

Supplement: Supplementary file 1 [file ijms-25-05975-s001.zip › ijms-3037026-supplementary/Figure S3.tif]
